# Effect of bradykinin on TGF-β1-induced retinal pigment epithelial cell proliferation and extracellular matrix secretion

**DOI:** 10.1186/s12886-016-0373-3

**Published:** 2016-11-10

**Authors:** Wenting Cai, Qingquan Wei, Qingyu Liu, Chengda Ren, Junling Liu, Ruiling Zhang, Mengmei He, Qianyi Wang, Yaru Du, Jing Yu

**Affiliations:** 1 Department of Ophthalmology, Shanghai Tenth People’s Hospital, Tongji University, School of Medicine, Shanghai, China; 2 Nanchang University, Jiangxi, China; 3 Department of Ophthalmology, The 180th Hospital of Chinese People’s Liberation Army, Quanzhou, Fujian province China

**Keywords:** Proliferative vitreoretinopathy, Retinal pigment epithelium, Bradykinin, Transforming growth factor-β1, Extracellular matrix

## Abstract

**Background:**

To evaluate the effect of bradykinin (BK) on TGF-β1-induced retinal pigment epithelial (RPE) cell proliferation and extracellular matrix secretion and to elucidate the relationship between BK and the Erk/Akt signaling pathway.

**Methods:**

The effects of BK on TGF-β1-induced RPE cell proliferation were examined via CCK-8 assay. Cell culture supernatant collagen I concentrations were measured via ELISA. Fibronectin (Fn), matrix metalloproteinase-2 (MMP-2) and MMP-9 mRNA and protein expression levels were measured via q-PCR and Western blotting, respectively. Changes in Akt/Erk phosphorylation induced by BK and HOE-140 were evaluated via Western blotting.

**Results:**

TGF-β1 stimulated ARPE-19 cell proliferation, which was inhibited by BK, whose effects were inhibited by HOE-140. BK inhibited TGF-β1-induced collagen I, Fn and MMP-2 secretion in RPE cells, and these effects were inhibited by HOE-140. BK also inhibited TGF-β1-induced Akt phosphorylation in RPE cells, and these effects were blocked by HOE-140. BK had no significant effect on Erk-mediated signaling.

**Conclusions:**

The findings from this study indicate that BK could be novel therapeutic targets for the treatment of PVR.

## Background

Proliferative vitreoretinopathy (PVR) is the most common cause of failed repair of rhegmatogenous retinal detachment [1]. The major feature of PVR is the formation of a subretinal or epiretinal membrane (ERM) that consists of retinal pigment epithelial (RPE) cells, fibroblasts, glial cells, and macrophages, as well as extracellular matrix [2]. Besides, concomitant inflammatory cells are involved in the formation of proliferative membrane [3]. The pathogenesis of PVR includes migration of cells, proliferation of migrating cells, membrane development, contraction, extracellular collagen production and creation of fixed folds in the retina [4].

Under physiological conditions, RPE cells exist in a resting state and remain in G_0_ phase, which maintains normal retinal function. Human RPE (hRPE) cells are frequently used in in vitro studies to investigate RPE-related disesases, as well as specific clinical treatments for these diseases. Thus, the hRPE cell line ARPE-19 was used in our study [2, 5, 6].

Previous studies have reported that abnormal ECM deposition and cell proliferation increase the likelihood of PVR development, and the newly formative ECM promotes membrane contraction. Therefore, preventing abnormal ECM deposition and cell proliferation may prevent PVR development [3, 7]. Collagen is the most abundant protein in the human body and comprises several different subtypes. Types I and III contribute to PVR membrane formation [8], which results in RPE cell phenotype changes, as well as RPE cell proliferation, migration and deformation [9]. Fibronectin (FN), laminin (LN) and vitronectin (VN) are the major components of non-collagenous glycoproteins. Previous studies have shown that FN influences specific RPE cell behaviors, including trans-differentiation, proliferation, migration, adhesion, contraction and cytoskeletal formation [10].

The balance between ECM production and degradation is tightly regulated, and matrix metalloproteinases (MMPs) are associated with the degradation of collagen and other ECM proteins [11]. Other studies have shown that MMP activity correlates with PVR membrane formation [12]. Some studies have shown that MMP-1 exists in the normal retina, while MMP-2 and MMP-9 exist in the epiretinal and subretinal membranes and facilitate cell migration to the vitreous cavity in the setting of early PVR [13, 14]. Gonzalez-Avila noted obvious increases in MMP-2 and MMP-9 expression in the subretinal fluid of PVR patients [15]. MMPs play an important role in regulating ECM remodeling.

In PVR, ECM synthesis, secretion and degeneration are stimulated not only by MMPs but also by growth factors, especially TGF-β1. Dvashi et al. find that TGF-β1 Induces Transdifferentiation of RPE Cell via TAK1 pathway [16]. TGF-β1 stimulates RPE cell type I, III and IV collagen expression [17, 18]. Previous studies have found that inhibiting TGF-β1 expression may prevent PVR progression [19]. TGF-β1 plays a vital role in PVR formation, as it regulates cell proliferation, promotes ECM synthesis and induces ECM deposition at wound sites, resulting in scarring and fibrosis [8, 20].

The kallikrein-kinin system (KKS) is one of the main pressure-releasing systems in the human body, which comprises kininogen (KNG), kinin, kininase, kallikrein, kallikrein and kinin receptors, also plays a role in PVR progression [21]. Some studies found that KKS had the pro-inflammatory effects [22] and increased the permeability of the retinal microvasculature [23]. But the effect of BK on the process of PVR is controversial. So we focus on KKS, which modulates several important functions, in order to find new therapies to PVR. Bradykinin (BK) is the primary determinant of KKS activity [24]. Our previous studies have shown that KNG1 is localized in the vitreous body and that its expression is positively correlated with PVR severity [25, 26]. High levels of BK expression have been detected in the serum, vitreous body and retina of PVR rats, which indicates that BK may be associated with PVR development [21]. Nelly Blaes reported that BK inhibits TGF-β-induced collagen production by activating the bradykinin B2 receptor (B2R) [27]. Other studies have found that BK inhibits growth factor-induced cell proliferation and ECM secretion [28]. However, the effects of BK on cultured RPE cell proliferation and the mechanism underlying its effects on TGF-β1 remain unknown. Therefore, we investigated the effect of BK on TGF-β1-induced RPE cell proliferation and extracellular matrix secretion.

## Methods

### Reagents and antibodies

BK and anti-MMP-2 antibodies were obtained from Abcam (Cambridge, UK). The BK B2 receptor-specific antagonist, HOE-140, was purchased from Shanghai Top-Peptide Biotechnology Company (Shanghai, China). Recombinant human TGF-β1 was obtained from PeproTech Inc. (New Jersey, USA). Antibodies to fibronectin, Akt, and phosphorylated Akt (p-Akt) were purchased from Bioworld (Minnesota, USA), and antibodies to p-Erk1/2 and anti-MMP-9 were obtained from Cell Signaling Technology (Boston, USA).

### Cell culture and general experimental methods

ARPE-19 cells (ICell Bioscience, China) were used for the experiments and were cultured in 10 % fetal bovine serum (FBS)-supplemented (Gibco, USA) DMEM/F12 culture medium (Hyclone, USA) at 37 °C in a humidified atmosphere of 5 % CO_2_. The experiments were performed using cultured ARPE-19 cells (passages two through four) that were grown until they exhibited a hexagonal shape and no visible pigmentation. Upon reaching 60–70 % confluence, the ARPE-19 cells were cultured for 24 h in FBS-free DMEM/F12 culture medium to achieve cell synchronization. We treated the cells with four different interventions. The cells were incubated with 0.1, 0.5, 2.5, 10, 12.5 ng/ml TGF-β1 and 0.1, 1, 10, 100, 1000 nM BK for 24 and 48 h. Cell Counting Kit-8 (CCK-8) (Beijing Zoman Biotechnology Co., Ltd., China) was applied to detect cell proliferation. In addition, after the ARPE-19 cells were pre-incubated with various concentrations (0.1, 1, 10, 100, 1000 nM) of BK for 30 min, they were coincubated with 10 ng/ml TGF-β1 for 24 and 48 h so that we could observe the effect of BK on TGF-β1-induced ARPE-19 cell proliferation. We pre-incubated the cells with 1 μM HOE-140 for 30 min before treating them with or without exogenous TGF-β1 and BK.

### Cell proliferation detection by CCK-8

ARPE-19 cells were diluted and suspended in complete culture medium containing 10 % FBS before being plated at a density of 2000 cells/well (100 μl/well) in 96-well plates for 24 h. The cells were starved in basal medium without FBS for 12 h to achieve synchronization. After being treated with the above different interventions, the cells were cultured for 24 and 48 h and then coincubated with 10 μl of CCK-8/well at 37 °C in the dark for 3 h. Optical density (OD) was detected at 450 nm using a microplate reader. Each group had five parallel wells, and detection was repeated three times per group to obtain mean values, which were used for subsequent statistical analyses.

### Type I collagen ELISA

Cell supernatant type I collagen (Col I) secretion was assessed using a human Col I enzyme-linked immunosorbent assay (ELISA) kit (Shanghai Senxiong Company, China), according to the manufacturer’s instruction. Cells were seeded in 6-well plates at a density of 2 × 10^5^ cells/well and treated with five different interventions. The control group was incubated with phosphate-buffered saline (PBS), the BK group was incubated with 1 nM BK, the TGF-β1 group was incubated with 10 ng/ml TGF-β1, and the BK and TGF-β1 group was pre-incubated with 1 nM BK for 30 min before being treated with 10 ng/ml TGF-β1. The HOE-140, BK and TGF-β1 group was pre-incubated with 1 μM HOE-140 and 1 nM BK for 30 min each before being treated with 10 ng/ml TGF-β1. All groups were cultured for 48 h after the above interventions. Their supernatants were subsequently collected and centrifuged. Col I concentrations were measured at 492 nm and calculated from a standard curve.

### Q-PCR for fibronectin, MMP-2, and MMP-9 mRNA expression measurements

q-PCR was used to measure mRNA expression. Total RNA was extracted using Trizol (Invitrogen, USA). After incubation, the cells were washed with PBS and treated with 1 ml of Trizol before being centrifuged for 5 min. Chloroform (Shanghai Generay Biotech Co., Ltd, China) was added (200 μl of chloroform/ml Trizol) to the reaction tube, which was shaken vigorously, incubated for 5 min and centrifuged at 12000 rpm for 15 min. The aqueous fluid was then removed from the tube and placed in another centrifuge tube, mixed with isopropanol (Shanghai Generay Biotech Co., Ltd, China) (500 μl isopropanol/ml Trizol), incubated for 10 min, and centrifuged at 12000 rpm for 10 min for RNA precipitation. The mixture was then dried using anhydrous ethanol before being treated with 30 μl of DEPC (Sigma, USA) water to dissolve the RNA precipitate. The RNA concentration was subsequently detected via spectrophotometry with an A260/280 ratio of 1.8–2.0.

cDNA was synthesized via RT-PCR (TaKaRa, Japan), according to the manufacturer's protocol. cDNA was amplified with specific primers. GAPDH primers were used as controls. The nucleotide sequences of fibronectin, MMP-2, MMP-9 were obtained from GenBank and used to design specific primers (synthesized by Sangon Corporation, Shanghai, China), the sequences of which are shown in Table 1. A total of 0.5 μl of reverse-transcribed cDNA was subjected to PCR in the presence of 10 μl of PCR mix and 0.5 μl of sense and antisense primers. q-PCR was used to detect Fn, MMP-2, and MMP-9 mRNA expression.

**Table 1:** RT-qPCR primers

Primer name	Primer sequence (forward/reverse)
GAPDH	5’-CTGGGCTACACTGAGCACC-3’ 5’-AAGTGGTCGTTGAGGGCAATG-3’
Fibronectin	5’- CGGTGGCTGTCAGTCAAAG-3’ 5’-AAACCTCGGCTTCCTCCATAA-3’
MMP-2	5’-TACAGGATCATTGGCTACACACC-3’ 5’-GGTCACATCGCTCCAGACT-3’
MMP-9	5’-TGTACCGCTATGGTTACACTCG-3’ 5’-GGCAGGGACAGTTGCTTCT-3’

Relative mRNA expression levels were calculated as the difference between target and reference gene (GAPDH) expression levels using the ΔCT method. Real-time PCR was performed in triplicate for each gene, including GAPDH. The threshold cycles at which exponential increases in PCR product amplification were detected were used for quantification.

### Western blot analysis

Fibronectin, MMP-2 and MMP-9 protein expression levels were detected by Western blotting. Cold radio-immunoprecipitation assay (RIPA) buffer and phenylmethylsulfonyl fluoride (PMSF: a protease inhibitor) (Shanghai Beyotime Biotech Co., China) were used to lyse the cells, which were washed twice with PBS. A cell scraper was used to gather the cell lysates, which were subsequently transferred to Eppendorf tubes and centrifuged for 30 min at 12,000 rpm (4 °C) to obtain the supernatants. A BCA protein assay kit (Shanghai Beyotime Biotech Co., China) was used to determine protein concentrations. Protein samples (30 μg protein/well) were electrophoresed via sodium dodecyl sulfate polyacrylamide gel electrophoresis (SDS-PAGE) and then transferred to polyvinylidene difluoride (PVDF) membranes, which were sequentially blocked in Tris-buffered saline containing 5 % milk for 1 h at 37 °C, incubated with antibodies to fibronectin, MMP-2 and MMP-9 overnight, washed, and then incubated with the appropriate secondary antibody for 1 h at 37 °C. Band densities were quantified using Image J software.

Akt, Erk 1 and 2 expression levels were measured by Western blotting, as described above. The cells were incubated with BK, TGF-β1 and the BK receptor antagonist HOE-140 to determine the effect of HOE-140 on Akt and Erk 1 and 2 phosphorylation.

### Statistical analysis

Statistical analyses were performed with SPSS 19.0 software. All measurement data were representative of at least 3 separate experiments, are expressed as the mean ± standard deviation (SD) and were compared between two groups using Student’s *t*-test. For multiple comparisons, the results were analyzed using one-way ANOVA. *P*-values less than 0.05 were considered significant.

## Results

### The effects of TGF-β1, BK and HOE-140 on APRE-19 cell proliferation

#### TGF-β1 stimulated cell proliferation

**Fig. 1:**
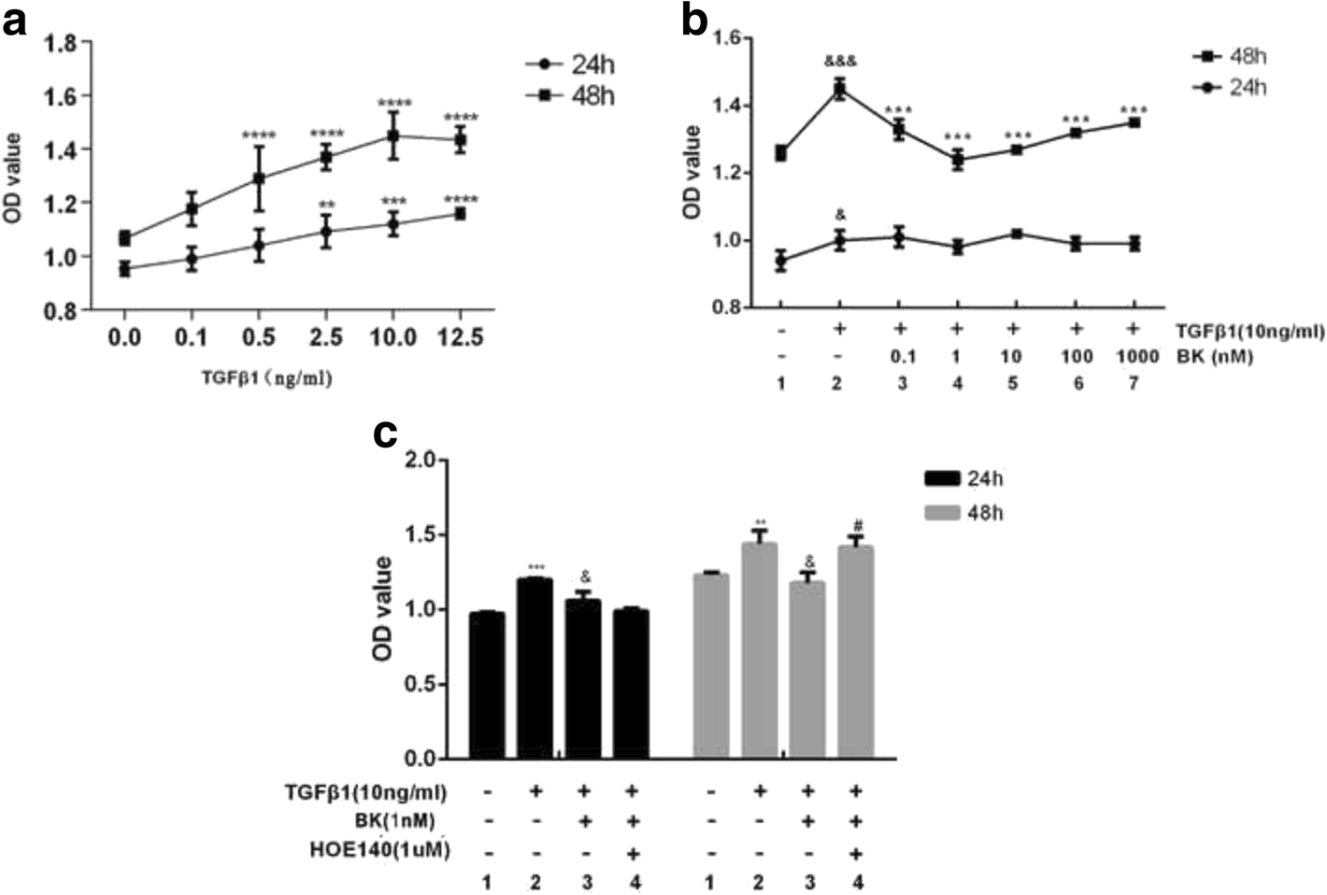
**a** Effect of 24 and 48 h of TGF-β1 stimulation on RPE cell proliferation. **P < 0.01, ***P < 0.001, ****P < 0.0001 versus the OD value of cells cultured without TGF-β1. **b** Effect of BK on TGF-β1-induced cell proliferation. & *P* < 0.05, &&& *P* < 0.001 versus without TGF-β1 and BK. ***P < 0.001 versus TGF-β1 without BK. **c** Effect of HOE-140 on the inhibitory effects of BK. **P < 0.01, ***P < 0.001 versus without addition, &*P* < 0.01 versus with TGF-β1 alone, ^#^ *P* < 0.01 versus with TGF-β1 and BK

TGF-β1 promoted cell proliferation at 24 and 48 h. Cell proliferation was positively correlated with TGF-β1 concentrations, with the exception of 12.5 ng/ml. Cell proliferation was significantly increased after 48 h of incubation compared with 24 h of incubation (Fig. 1a).

#### BK had no direct effect on cell proliferation

ARPE-19 cells were treated with different BK concentrations for 24 h and 48 h; however, no significant differences in OD values were noted between the BK-treated group and the control group (0 nM BK) (*P > 0.05*).

#### BK inhibited TGF-β1-induced cell proliferation through B2R activation

Our previous study demonstrated that 10 ng/ml TGF-β1 increases cell proliferation (*P < 0.05*). ARPE-19 cells were pre-incubated with different BK concentrations for 30 min. BK inhibited TGF-β1-induced cell proliferation, especially at a concentration of 1 nM (Fig. 1b). Before the cells were pre-incubated with 1 nM BK, they were incubated with 1 μM HOE-140 for 30 min. HOE-140 prevented the inhibition TGF-β1-induced cell proliferation facilitated by BK (*P = 0.003*, Fig. 1c).

### The effects of BK, HOE-140 and TGF-β1 on ARPE-19 cell collagen 1 section

**Fig. 2:**
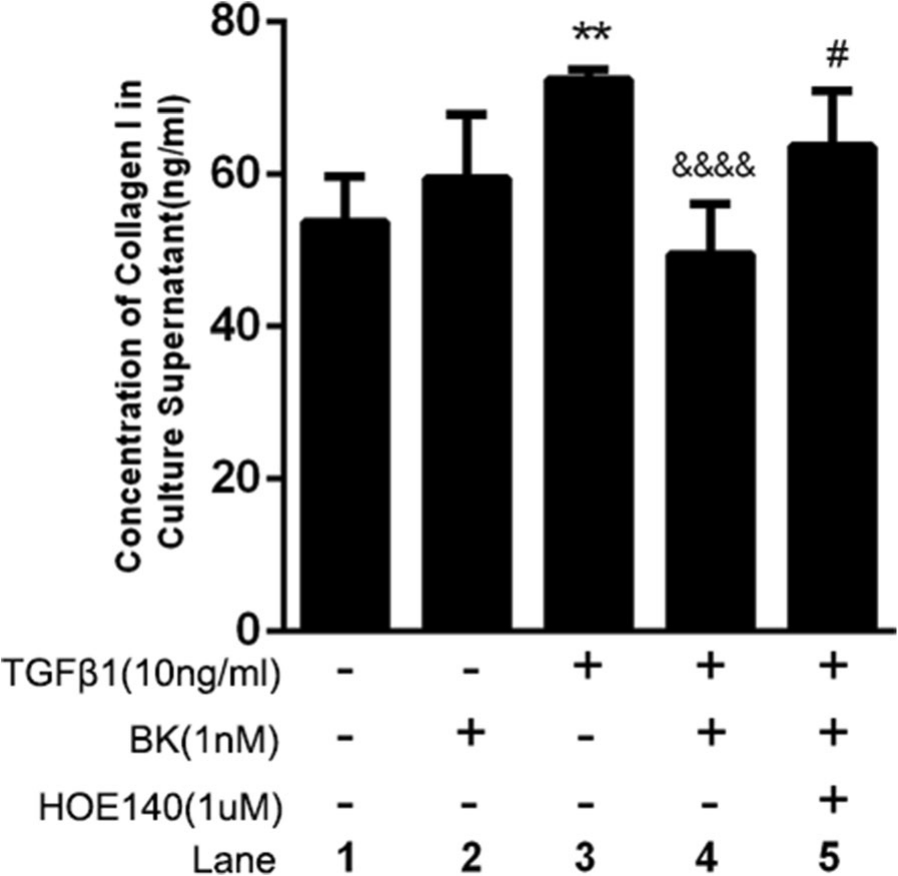
Effect of BK, HOE-140 and TGF-β1 on Col I secretion in RPE cells. ** *P* < 0.01 versus without addition, ****P < 0.0001 versus with TGF-β1 alone, *P < 0.05 versus with BK and TGF-β1

A single application of 10 ng/ml TGF-β1 promoted Col I secretion in ARPE-19 cells (*P = 0.005*), while a single application of BK had no significant effect on Col I secretion. TGF-β1-induced Col I secretion was significantly inhibited (compared to PBS alone group) when the cells were pre-incubated with BK before being treated with TGF-β1 (*P < 0.0001*). HOE-140 blocked the effects of BK on TGF-β1-induced Col I secretion (*P < 0.05*), as shown in Fig. 2.

### The effects of BK, HOE-140 and TGF-β1 on Fn, MMP-2 and MMP-9 mRNA expression in ARPE-19 cells

**Fig. 3:**
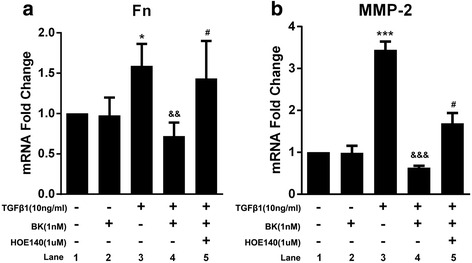
Effect of BK, HOE-140 and TGF-β1 on fibronectin and MMP-2 mRNA expression in RPE cells. *P < 0.05, *** *P* < 0.001 versus without addition. && *P* < 0.01, &&& *P* < 0.001 versus with TGF-β1 alone. ^#^ *P* < 0.05 versus with BK and TGF-β1

TGF-β1-treated ARPE-19 cells exhibited increased Fn and MMP-2 mRNA expression compared with control cells (both *P < 0.05*); however, a single application of BK had no significant effects on Fn or MMP-2 mRNA expression (*P > 0.05*). BK administration prior to TGF-β1 treatment inhibited TGF-β1-induced increases in Fn and MMP-2 mRNA expression (both *P < 0.05*). HOE-140 blocked the effects of BK on TGF-β1-induced Fn and MMP-2 mRNA expression (both *P < 0.05*), as shown in Fig. 3. The expression levels of MMP-9 in each of the six groups were low; thus, comparisons among these groups were not performed.

### The effects of BK, HOE-140 and TGF-β1 on Fn and MMP expression in ARPE-19 cells

**Fig. 4:**
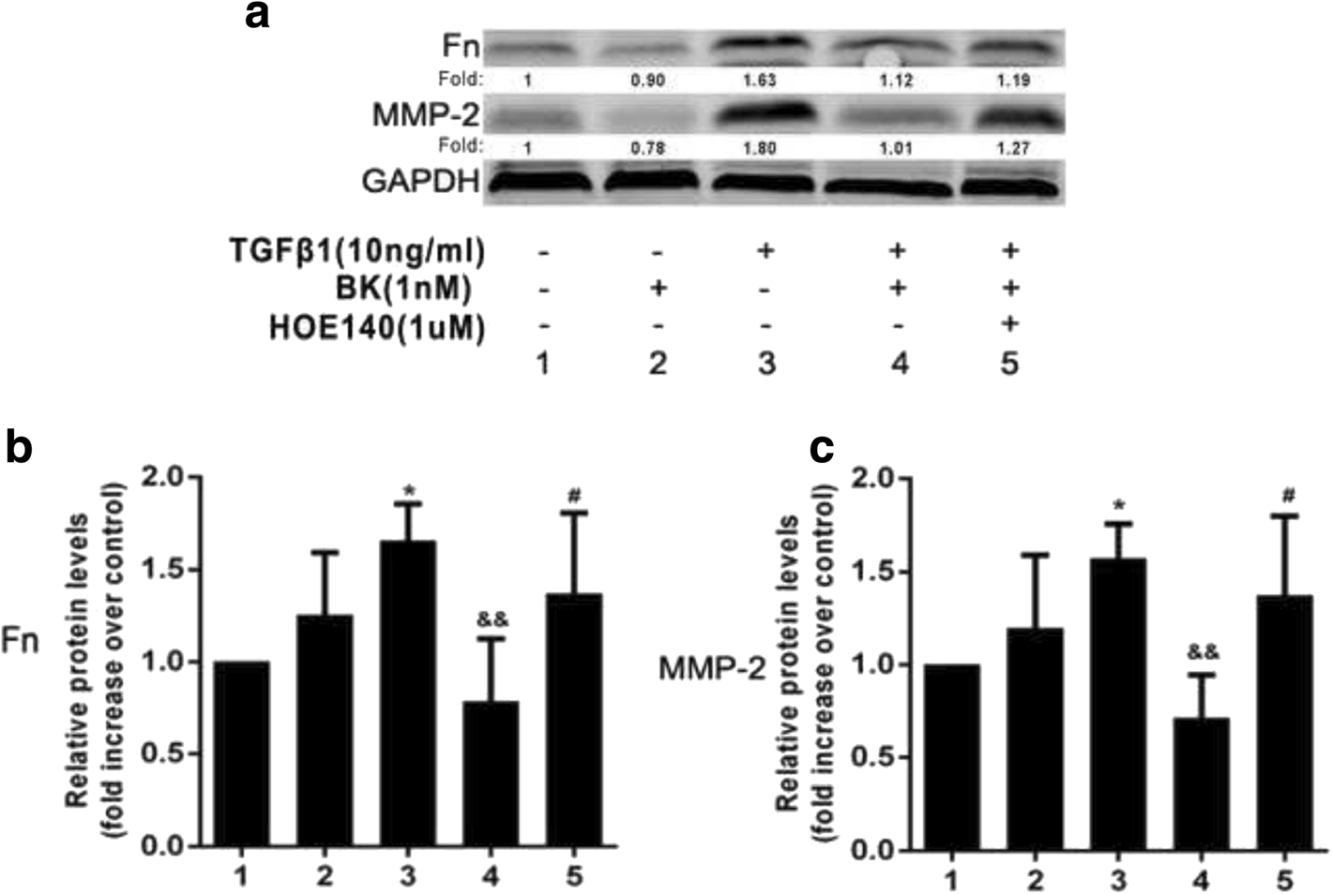
Effect of BK on TGF-β1-induced Fn and MMP-2 protein expression in RPE cells. **a** Western blotting results for Fn and MMP-2 protein expression in five groups. **b**/**c** TGF-β1 promoted Fn and MMP-2 secretion, which was inhibited by BK. HOE-140 blocked the inhibitory effects of BK. *P < 0.05 versus without addition. &&*P* < 0.01 versus with TGF-β1 alone. ^#^ *P* < 0.05 versus with BK and TGF-β1

Western blotting was performed to verify the abovementioned q-PCR results. As shown in Fig. 4, TGF-β1 promoted Fn and MMP-2 secretion by ARPE-19 cells, BK inhibited Fn and MMP-2 section, and HOE-140 blocked these effects (all *P < 0.05*). Therefore, the Western blotting results were consistent the abovementioned q-PCR results.

### The effects of BK on Akt and Erk1/2 phosphorylation

#### BK promoted Akt and Erk1/2 phosphorylation

**Fig. 5:**
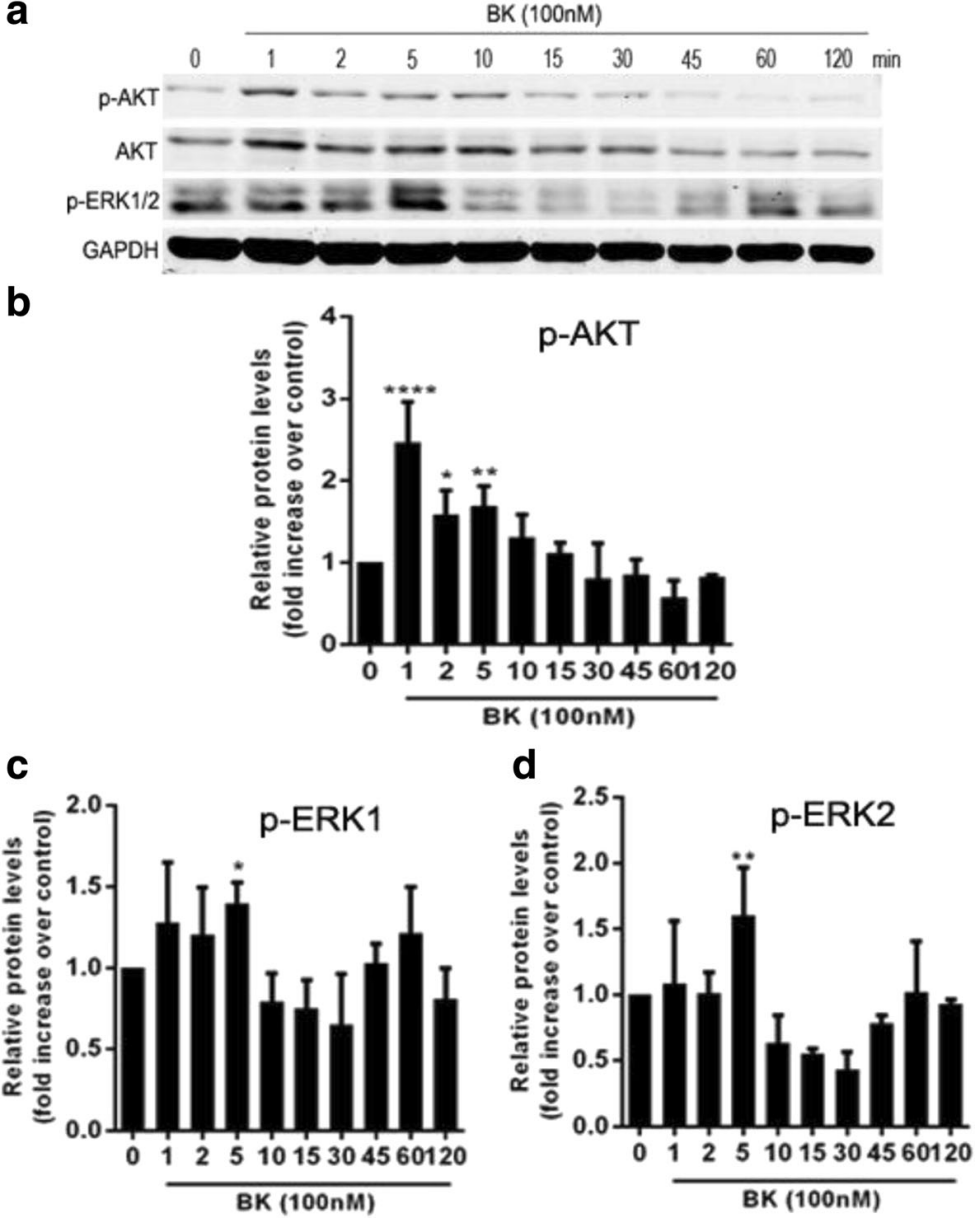
Effect of 100 nM BK on Akt and Erk1/2 phosphorylation in RPE cells during different periods. **a** Western blotting results for Akt and Erk1/2 phosphorylation during different periods. **b** Akt phosphorylation levels increased most significantly after 1 min of BK stimulation. **c**/**d** Erk1/2 phosphorylation levels increased most significantly after 5 min of BK stimulation. *P < 0.05, ** *P* < 0.01, **** *P* < 0.0001 versus with 0 min

**Fig. 6:**
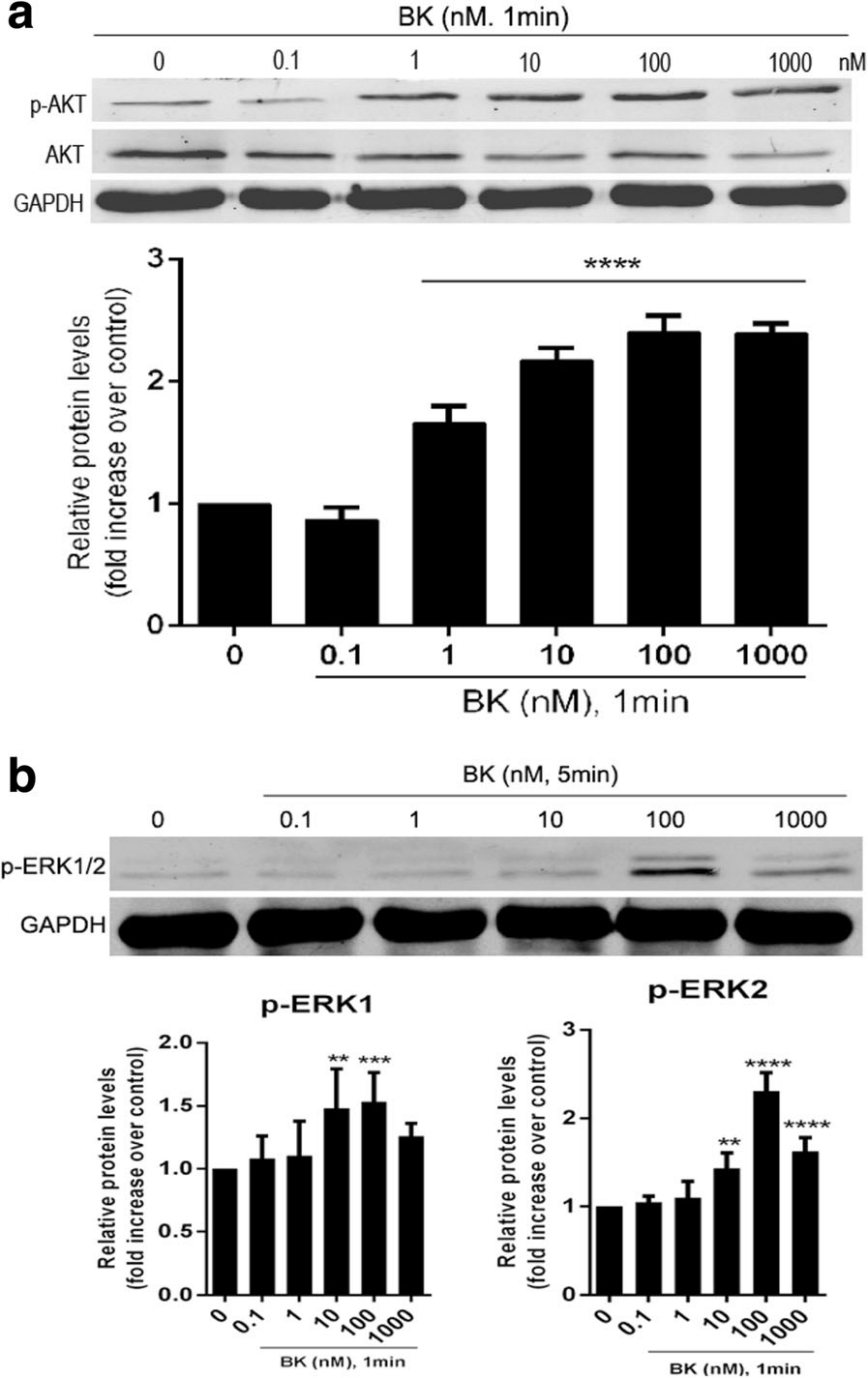
Effect of different concentrations of BK on Akt and Erk1/2 phosphorylation in RPE cells. **a** Akt phosphorylation levels increased most significantly after 1 min of BK stimulation. **b** Erk1/2 phosphorylation levels increased most significantly after 5 min of BK stimulation. *P < 0.05, **P < 0.01, ***P < 0.001, ****P < 0.0001 versus with 0 nM BK

BK promoted Akt and Erk1/2 phosphorylation in RPE cells. Akt phosphorylation levels increased most significantly after 1 min of BK stimulation, and Erk phosphorylation levels increased most significantly after 5 min of BK stimulation (both *P < 0.05*), as shown in Fig. 5. BK increased Akt and Erk1/2 phosphorylation levels over 1 and 5 min in a concentration-dependent manner in treated ARPE-19 cells compared with control cells (Fig. 6a, b).

#### BK inhibited TGF-β1-induced Akt phosphorylation

**Fig. 7:**
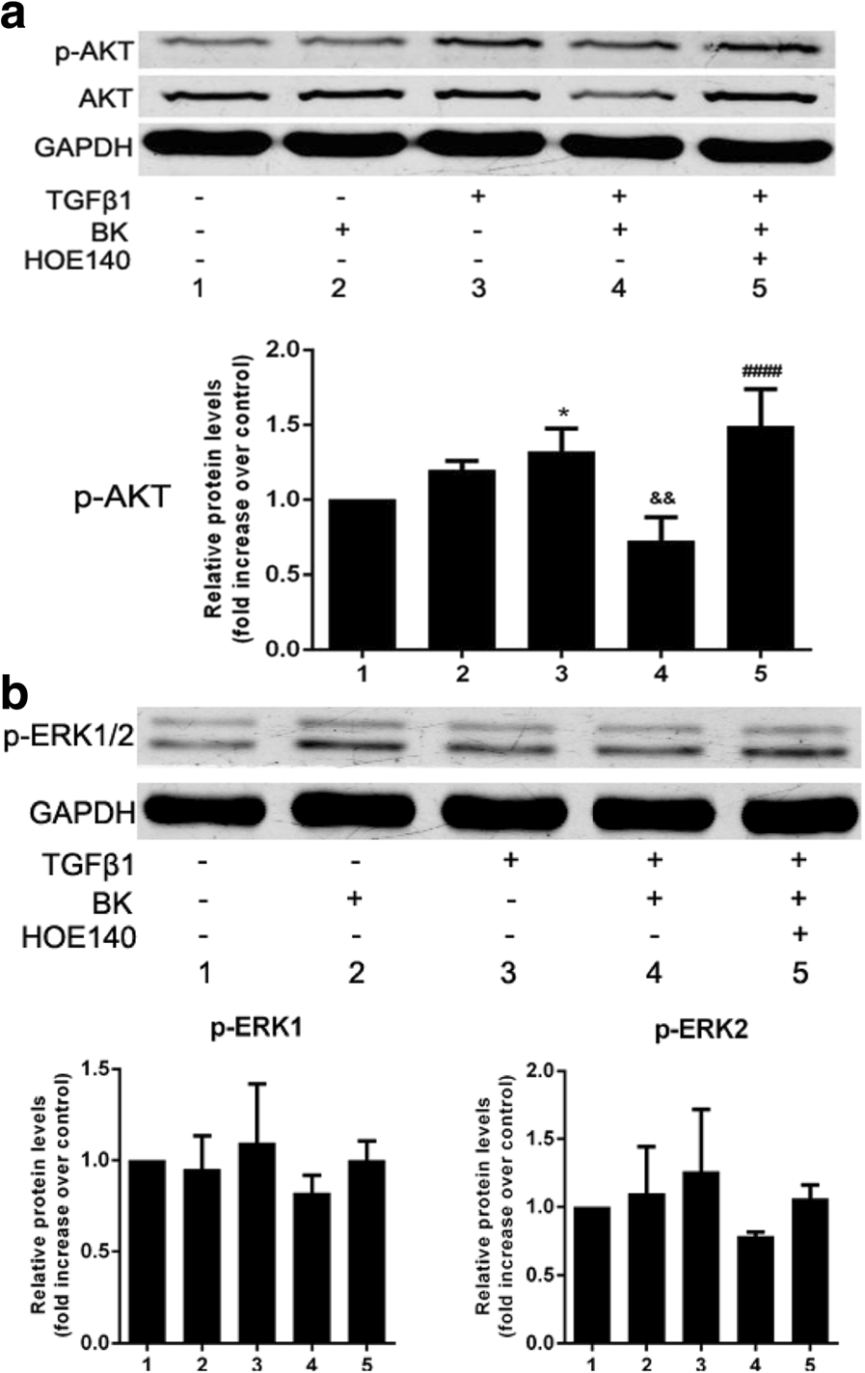
**a** Effect of BK on TGF-β1-induced Akt phosphorylation in RPE cells. **a** TGF-β1 facilitated significant increases in Akt phosphorylation levels in treated RPE cells compared to control cells; however, this effect was inhibited via incubation with BK before TGF-β1 treatment but could be blocked by HOE-140. **b** Neither BK nor HOE-140 had an effect on TGF-β1-induced Erk1/2 phosphorylation. *P < 0.05 versus without addition, && *P* < 0.01 versus with TGF-β1 alone. ^####^ *P* < 0.0001 versus with BK and TGF-β1

TGF-β1 significantly increased Akt phosphorylation levels in treated ARPE-19 cells compared with control cells (*P < 0.05*); however, pre-incubation with BK inhibited these effects, as shown in Fig. 7a. However, BK administration had no significant effects on TGF-β1-induced Erk1/2 phosphorylation (*P > 0.05*), as shown in Fig. 7b.

#### BK inhibited TGF-β1-induced Akt phosphorylation via the BK B2 receptor

To determine the mechanism underlying BK activity, cells were treated with HOE-140, a BK B2 receptor antagonist, before being treated with BK and TGF-β1. HOE-140 significantly inhibited the effects of BK on TGF-β1-induced Akt phosphorylation, indicating that the BK B2 receptor plays a role in inhibiting Akt phosphorylation (Fig. 7a). HOE-140 did not affect Erk1/2 phosphorylation (Fig. 7b).

## Discussion

Proliferative vitreoretinopathy (PVR) is a complication that can follow rhegmatogenous retinal detachments. Proliferative membrane contraction can result in reduced vision or blindness [1]. ECM deposition and cell proliferation are associated with PVR development, however, the mechanism underlying the relationship between ECM deposition and cell proliferation and PVR development is unclear. The KKS is one of the main pressure-releasing systems in the human body, and BK is the primary determinant of KKS activity and thus plays a vital role in regulating inflammation, cell proliferation, and matrix hyperplasia. The present study attempted to elucidate the mechanism by which BK protects against PVR development. Multiple types of cells and cytokines are involved in the pathogenesis of PVR, and TGF-β1-induced cell proliferation is closely related to PVR development.

Many scholars believe that inhibiting cell proliferation may prevent or delay PVR onset and progression. In this study, we performed CCK-8 assay to detect RPE cell proliferation, as this assay is an indicator of cell viability. We determined that TGF-β1 stimulates RPE cell proliferation and that although BK exerts no direct effects on cell proliferation, it can affect cell proliferation by significantly inhibiting the effects of TGF-β1. In addition, HOE-140, a bradykinin B2 receptor antagonist, blocked the effects of BK on TGF-β1. Therefore, this study demonstrated that BK regulates TGF-β1-induced RPE cell proliferation by activating the BK B2R.

TGF-β1 is an important regulator of ECM synthesis and degradation, as well as fibrosis [29, 30]. Connor showed that PVR patients exhibit significant increases in their vitreous fluid TGF-β concentrations and that these increases are directly proportional to PVR severity [31]. Baudouin noted increased TGF-β1 concentrations in subretinal fluid samples that were collected during different PVR periods. These findings indicate that TGF-β1 plays a role in PVR development and that inhibiting TGF-β1 expression may prevent PVR development [32]. Obeta reported that TGF-β1 activates Smad-dependent and Smad-independent signaling by binding TGF-β receptors. The Smad-independent signaling pathways comprise the PI3K-Akt, mitogen-activated protein kinase (MAPK), and JNK/p38 pathways, as well as other pathways [33]. The extracellular signal-related kinase1/2 (Erk1/2) signaling pathway is one of the most important signaling pathways associated with MAPK activity, which can be activated via phosphorylation cascade stimulation to regulate cell proliferation and differentiation [34]. PI3K plays a role in cell proliferation and differentiation and has been shown to regulate proto-oncogene expression. PI3K/Akt (also known as protein kinase B, PKB) signaling plays an important role in cell proliferation and survival [35].

Previous studies have shown that BK increases vascular smooth muscle cell proliferation by activating the Erk signaling pathway and that BK may also play a role in collagen synthesis and secretion by inhibiting TGF-β-induced Akt phosphoylation [27].

In the present study, ARPE-19 cells were stimulated by TGF-β1 and then treated with HOE-140 to determine the effect of BK on TGF-β1-induced ECM and MMP secretion by ARPE-19 cells, elucidate the mechanism underlying this effect, and clarify the relationship between BK and Akt/MAPK (Erk1/2) signaling. We found that BK inhibited TGF-β1-induced ECM deposition and MMP secretion. Moreover, TGF-β-induced Akt phosphorylation was also inhibited by BK. This inhibition was blocked by HOE-140, indicating that BK inhibits the effects of TGF-β1. The BK B2R is a G protein-coupled receptor (GPCR), and the mechanism underlying the effects of BK may involve protein-protein interactions between GPCRs and TGF-β receptors. Additional in-depth studies must be performed to identify the intracellular signaling pathways and specific protein-protein interactions associated with the effects of BK.

This study had some limitations. First, the KKS system is complicated. Although BK is the primary determinant of KKS activity, other substances in the KKS system may also have an impact on collagen formation in RPE cells. Second, our study involved only cultured RPE cells. We did not establish an animal model. Third, our study explored the effects of BK on TGF-β1-induced RPE cell proliferation; however, in the process of PVR, there are many growth factors such as transforming growth factor, hepatocyte growth factor, insulin-like growth factor and so on. A variety of growth factors are involved in the process of PVR, cell function changes stimulated by different growth factors may lead to changes in downstream effector molecules. Thus, additional studies must investigate the effects of BK under different conditions and the mechanism underlying these effects.

In conclusion, this study showed that BK affects cell proliferation and ECM secretion and has shed new light on the mechanisms underlying PVR development, which may facilitate the development of new therapies for this disease.

## Conclusions

BK exerts its effects by binding the BK B2R in TGF-β1-treated RPE cells and was found to inhibit TGF-β1-induced cell proliferation. BK inhibited TGF-β1-induced Fn, collagen I and MMP-2 secretion and Akt phosphorylation. The findings from this study indicate that BK could be novel therapeutic targets for the treatment of PVR.

## Abbreviations

BK: Bradykinin
CCK-8: Cell counting Kit-8
ECM: Extracellular matrix
ELISA: enzyme-linked immunosorbent assay
Fn: Fibronectin
KKS: Kallikrein-kinin system
KNG: Kininogen
LN: Laminin
MMP-2: Matrix metalloproteinase-2
OD: Optical density
PBS: Phosphate-buffered saline
PMSF: Phenylmethylsulfonyl fluoride
PVR: Proliferative vitreoretinopathy
RIPA: Radio-immunoprecipitation assay
RPE: Retinal pigment epithelial
VN: Vitronectin
